# Impact of Immune Parameters and Immune Dysfunctions on the Prognosis of Patients with Chronic Lymphocytic Leukemia

**DOI:** 10.3390/cancers13153856

**Published:** 2021-07-30

**Authors:** Candida Vitale, Elia Boccellato, Lorenzo Comba, Rebecca Jones, Francesca Perutelli, Valentina Griggio, Marta Coscia

**Affiliations:** 1 University Division of Hematology, A. O. U. Città della Salute e della Scienza di Torino, Via Genova 3, 10126 Torino, Italy; candida.vitale@unito.it (C.V.); elia.boccellato@unito.it (E.B.); lorenzo.comba179@edu.unito.it (L.C.); rebecca.jones@unito.it (R.J.); francesca.perutelli@unito.it (F.P.); valentina.griggio@unito.it (V.G.); 2 Department of Molecular Biotechnology and Health Sciences, University of Torino, Via Nizza 52, 10126 Torino, Italy

**Keywords:** chronic lymphocytic leukemia, immune dysfunctions, prognosis

## Abstract

**Simple Summary:**

In chronic lymphocytic leukemia (CLL), immune alterations—affecting both the innate and adaptive immunity—are very common. As a clinical consequence, patients with CLL frequently present with autoimmune phenomena, increased risk of infections and second malignancies. The aim of this review article is to present available data on CLL-associated alterations of immune parameters that correlate with known prognostic markers and with clinical outcome. Also, data on the impact of immune-related clinical manifestations on the prognosis of patients with CLL will be discussed.

**Abstract:**

Chronic lymphocytic leukemia (CLL) is characterized by a wide spectrum of immune alterations, affecting both the innate and adaptive immunity. These immune dysfunctions strongly impact the immune surveillance, facilitate tumor progression and eventually affect the disease course. Quantitative and functional alterations involving conventional T cells, γδ T cells, regulatory T cells, NK and NKT cells, and myeloid cells, together with hypogammaglobulinemia, aberrations in the complement pathways and altered cytokine signature have been reported in patients with CLL. Some of these immune parameters have been shown to associate with other CLL-related characteristics with a known prognostic relevance or to correlate with disease prognosis. Also, in CLL, the complex immune response dysfunctions eventually translate in clinical manifestations, including autoimmune phenomena, increased risk of infections and second malignancies. These clinical issues are overall the most common complications that affect the course and management of CLL, and they also may impact overall disease prognosis.

## 1. Introduction

Chronic lymphocytic leukemia (CLL) is a lymphoproliferative disease typically characterized by a wide spectrum of immune alterations, affecting both innate and adaptive immunity (reviewed in [1]). In addition to being a hallmark of the disease, CLL-associated immune dysfunctions strongly impact the immune surveillance, facilitate tumor progression and eventually affect the disease course.

**Figure 1:**
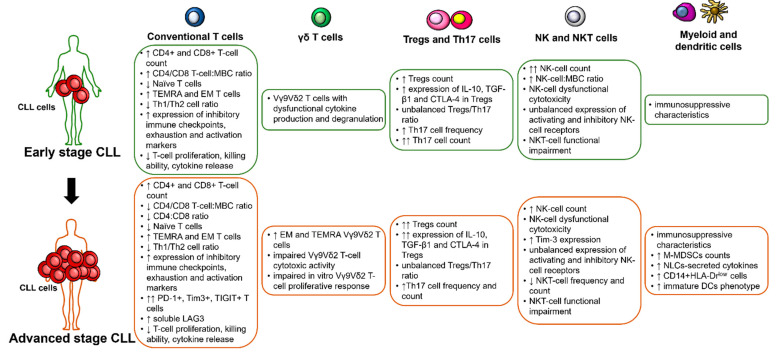
Summary of the main alterations characterizing immune cells of patients with early and advanced stage chronic lymphocytic leukemia. ↓ decreased; ↑ increased; ↑↑ particularly increased

The aim of this review article is to present available data on immune parameters which are altered in patients with CLL, and that can associate with other disease characteristics with a known prognostic relevance or that directly correlate with prognosis (Figure 1). In addition, we will discuss major immune-related clinical manifestations – such as autoimmunity, infections and second malignancies – that characterize CLL clinical course and may also impact on disease prognosis.

## 2. Specific Cellular and Humoral Immune Dysfunctions and Their Prognostic Impact in CLL

### 2.1. T Cells

#### 2.1.1. Conventional T Cells

T lymphocytes play a pivotal role in tumor immune-surveillance and immune response to infections. CD4+ T helper lymphocytes are the main coordinator of immune response via both cell-to-cell interactions and cytokine production, activating B lymphocytes and CD8+ cytotoxic T lymphocytes [reviewed in [2,3]]. In patients affected by CLL, CD4+ and CD8+ T lymphocyte numbers are increased [4,5,6,7], and several studies have explored a possible prognostic impact of conventional T-cell counts in this disease. The ratio of T cells:malignant monoclonal B cells (MBC) has been described as an independent predictor of time-to-first treatment (TTFT) in early stage CLL, with higher CD4:MBC and CD8:MBC ratios predicting longer overall survival (OS) [8]. Also, an indolent clinical course (i.e., a probability of OS at 10 years of 95%) has been described in a subgroup of patients characterized by elevated CD8:MBC ratio and the absence of CD38 expression on tumor cells [9].

In CLL, T cells undergo oligoclonal proliferation that is probably due to the chronic exposure to malignant B cells [10]. The expansion of CD8+ T lymphocytes outweighs that of CD4+, resulting in a reduced (also called inverted) CD4:CD8 ratio [5,6,7,11], which has been associated with advanced disease stages [6]. Interestingly, Apostolopoulos and colleagues showed that a lower CD4:CD8 ratio is also predictive of recurrent respiratory infections [12]. Consistently, several studies found that an inverted CD4:CD8 ratio is associated with shorter TTFT [7,13], progression-free survival (PFS) [14,15] and OS [13,14,15]. In the post-treatment setting, instead, it has been shown that following fludarabine-cyclophosphamide-rituximab (FCR) treatment, a higher CD4+ − but not CD8+ − T-lymphocyte count associate with a shorter PFS in patients maintaining a detectable minimal residual disease [16]. In this study, a thorough characterization of the CD4+ population revealed that it mostly consists of regulatory T cells (Tregs, CD25+CD127-FoxP3+), which are known to facilitate relapse and progression in CLL (see below).

The composition of T-cell differentiation subsets and Th1/Th2 cell distribution has also been extensively studied in patients with CLL. A reduction in naïve T cells and an increase in effector T lymphocytes and terminally differentiated memory T lymphocytes were consistently described [10,14,17,18]. Furthermore, most reports agree on the increase of Th1 lymphocytes in patients with CLL compared to healthy controls, whereas regarding Th2 cells the data are controversial [7,17,19,20,21]. Nevertheless, to our knowledge, to date there are no available data that clearly associate Th1 and/or Th2 cell subset expansion with CLL-related prognostic factors. Interestingly, Puzzolo and colleagues recently demonstrated that the treatment with the BTK ibrutinib induces a decrease in the Th1/Th2 cell ratio, which is more prominent in patients with unmutated immunoglobulin heavy chain variable region genes (IGHV) status and in those who achieve a complete response to therapy [22].

Th17 cells represent a subset of pro-inflammatory cells involved in inflammation and autoimmunity, which may play a dichotomous role in cancer [23]. Compared to healthy controls, patients affected by CLL have an increased frequency and absolute count of Th17 lymphocytes [17,21,24,25]. Concerning their role on disease evolution, a higher Th17-cell number has been correlated with early stages of the disease [26], and most [25,27,28] – but not all [26] – available data have reported a positive impact of high Th17-cell counts on OS.

In CLL, as in many other cancers, the tumor-mediated chronic antigenic stimulation of T cells also determines an increased surface expression of typical markers of activation [29,30], and affects their phenotypic and functional features. Leukemic B cells, through a direct cell-to-cell contact, modify the expression of genes involved in CD4+ and CD8+ T-cell differentiation and function [31], confirming the role of the malignant cells in nourishing a favorable pro-tumoral microenvironment. Several studies have reported that T lymphocytes from patients with CLL are primed for anergy because of their higher expression of immune-checkpoint molecules, such as CTLA-4 [32], PD-1 [18,33], LAG3 [34], Tim-3 [35], TIGIT [36], CD160 and CD244 [37], leading to a microenvironment characterized by reduced T-cell proliferation, killing ability and cytokine release. Of note, both CD8+ and CD4+ T-cell subsets show a complex co-expression of molecules indicating T-cell exhaustion (PD-1), senescence (KLRG-1) and activation (HLA-DR) [15].

Despite T-cell phenotypic and functional alterations have been extensively described in CLL, only few parameters have been reported to have a prognostic relevance. For example, higher soluble LAG3 levels in patients’ sera were found to be associated with progressive disease status and shorter TTFT [34]. These findings are possibly attributable to the higher expression of LAG3 mRNA detected in CLL cells with unmutated IGHV compared to mutated IGVH and normal B cells. Also, PD-1 upregulation was described in both CD4+ and CD8+ T cell subsets [18], and was associated with disease progression [14,15,17] and shorter TTFT [14]. Consistently, in a cohort of 80 patients with CLL, Palma et al. found that those with progressive disease had higher CD4+PD1+ and CD8+PD1+ T cells compared to both healthy controls and to non-progressive patients, with a bigger difference noted in previously treated patients [17]. Moreover, among previously treated patients, those with advanced disease stages showed an increased PD1+, Tim-3+ and TIGIT+ T-cell counts [35]. In line with these data, Jimenez et al. recently reported that patients who are in clinical progression present an accumulation of terminally exhausted effector CD8+ T cells, which are characterized by the co-expression of PD-1, CD244 and CD160 inhibitory receptors and by an altered gene expression profile [38]. Interestingly, these specific CD8+ T-cells alterations are possibly mediated by IL-10 released by leukemic cells [38]. Finally, Elston and colleagues examined in their cohort of 74 patients whether specific T-cell subsets could be associated with inferior clinical prognosis [15]. In multivariate analysis, higher percentages of circulating CD4+HLA-DR+PD1+ T-lymphocytes were associated with a shorter PFS.

#### 2.1.2. Gamma-Delta T Cells

γδ T lymphocytes are a subset of non-MHC-restricted T lymphocytes which account for 2 to 10% of circulating T cells in healthy subjects. γδ T cells have the ability to kill their targets via cytotoxic mechanisms and, thus, are involved in infection responses, autoimmunity and tumor immune surveillance [39].

The main circulating subset of γδ T lymphocytes is represented by Vγ9Vδ2 T cells, which recognize non-peptidic phosphoantigens, such as isopentenyl pyrophosphate and aminobisphosphonates, in a T cell receptor (TCR)-dependent fashion [40,41]. Vγ9Vδ2 T cells collected from patients with CLL show dysfunctional cytokine production and degranulation, resulting in less effective cytotoxicity toward tumor cells. Interestingly, a comparable dysfunctional phenotype is also inducible in Vγ9Vδ2 T cells yielded from healthy individuals when co-cultured with CLL cells, suggesting a leukemia-induced immune-dysfunction [42]. We have previously reported that the in vitro proliferative response of Vγ9Vδ2 T cells is significantly impaired in a subset of patients affected by CLL (low-responder, LR, patients), who also display an unbalanced Vγ9Vδ2 T-cell subset distribution in favor of effector memory and terminally differentiated effector memory cells [43]. The LR condition was associated with unmutated IGHV status and, most importantly, it was an independent predictor of shorter TTFT in multivariate analysis. These findings support the concept that tumor-induced chronic activation fosters the undesired accumulation of immune cells inadequate for an effective antitumor activity.

#### 2.1.3. Regulatory T Cells

Tregs are a fundamental CD4+ T-lymphocyte subset which prevents excessive immune activation and thus autoimmunity. In regards to hematological malignancies, Tregs may contribute to a pro-tumoral microenvironment, facilitating tumor progression [44]. Several studies demonstrated an increased count of circulating Tregs in CLL patients compared to healthy controls [45,46,47,48]. Interestingly, the expansion of Tregs is probably mediated by CD27-CD70 interactions and by a resistance to apoptosis, rather than by chronic antigenic stimulation [49]. Higher Treg numbers have been associated with increased tumor load and advanced stages of disease [45,50,51,52]. Specifically, Treg count progressively increases as the patients transition from an healthy status, to a monoclonal B cell lymphocytosis (MBL), and later to an overt and advanced CLL [53]; furthermore, Treg count is higher in patients with progressive disease compared to those with a stable disease [14,28,46,54]. Treg count has also been associated with CLL-related prognostic markers such as CD38 and ZAP70 expression [55], and in CLL, higher Treg absolute number and frequency correlate with shorter TTFT and OS [47,56,57]. Notably, different non-chemotherapy drugs such as thalidomide, lenalidomide, idelalisib and ibrutinib have shown the ability to restore or at least reduce Treg counts [58,59,60].

Beside the increased count, Tregs from CLL patients produce a larger amount of IL-10 and TGF-β1 and overexpress CTLA-4 compared to healthy controls [33,52,61]. Motta et al. showed in a cohort of 40 untreated patients with CLL, that CTLA-4 expression in CD4+CD25+ T cells is increased and correlates with advanced Rai stage, hypogammaglobulinemia, adverse cytogenetics and unmutated IGHV status [33].

Finally, the prognostic impact of Tregs in CLL has also been assessed in relation to other T-cell subsets. Specifically, a reduced Tregs/Th17 ratio due to Th17 number increase has been associated with autoimmune cytopenias [24]. Also, a higher Treg number has been correlated to dysfunctional Vγ9Vδ2 T lymphocytes in untreated CLL patients [43]. Taken together, all these observations suggest an active role of Tregs in the progression of the CLL.

### 2.2. Natural Killer and Natural Killer T Cells

#### 2.2.1. Natural Killer Cells

Natural Killer (NK) cells are fundamental components of the innate immune system and play an important role in antitumor immunity, being able to kill neoplastic cells without priming or prior activation [1,62]. However, the activity of NK cells, which is regulated by activating and inhibitory signals, is disrupted in malignant environments [63]. Specifically, in CLL, leukemic cells can interact directly with the host’s lymphocytes and secrete cytokines that alter the number, subset distribution and functions of NK cells [64].

Quantitative NK abnormalities have shown to impact both prognosis and therapeutic efficacy in CLL. Different groups have confirmed that, when compared to healthy donors, patients with CLL univocally present higher NK-cell count [8,9,65,66]. Interestingly, increased NK-cell count (>0.40 × 10^9^/L) corresponds to positive prognostic factors (i.e., early Rai or Binet stage, normal levels of β2-microglobulin, negativity of ZAP70 or CD38 expression, absence of *TP53* gene mutation, mutated IGHV status and absence of *ATM* deletion) [66]. Also, the NK-cell count does not seem to decrease with disease progression [65]. However, no correlation between NK-cell absolute number and TTFT was reported [8,9,66] and, in regard to OS prediction, only Wang et al. have correlated a shorter OS to a lower NK-cell count [66]. When examining NK-cell relative number, a higher NK:MBC ratio was associated to low-risk disease (i.e., early Rai stages and mutated IGHV status) [8,9], but only Palmer et al. correlated NK-cell relative number to a longer TTFT [8].

The lack of a strong prognostic correlation, even though NK-cell number is consistently increased in patients with CLL, may be attributable to the NK-cell dysfunctional cytotoxicity [65]. In line with this hypothesis, NK cells from patients with CLL have shown an impaired expression of the NKG2D costimulatory receptor and, consequently, a defective cytotoxic activity [65]. Along with quantitative defects, MBC can in fact also cause functional aberrations in the NK-cell compartment (briefly summarized in Table 1), thus affecting NK cell-mediated immune mechanisms that are innately able to recognize cancer cells and which are also often exploited by therapeutical approaches. Amongst the latter, we find anti-CD20 monoclonal antibody (mAb)-based therapies, whose anti-tumor activity is strongly linked to the NK cell-related antibody-dependent cell-mediated-cytotoxicity (ADCC). Multiple pieces of evidence support the hypothesis that NK-cell number and activity have a direct impact on the efficacy of specific treatment regimens. As an example, Vitale et al. showed that, in patients with CLL treated with a regimen containing the anti-CD20 mAb ofatumumab and lenalidomide, those who achieved a complete response had a higher baseline absolute number and a more preserved function of NK cells, as compared to those who did not respond [67]. Furthermore, in CLL the effectiveness of anti-CD20 mAb treatments is positively affected by recombinant human IL-15 and lenalidomide, which are able to induce NK-cell proliferation and to improve NK cells-mediated ADCC and cytotoxicity [65,66,68].

#### 2.2.2. Natural Killer T Cells

Another important player of the immune surveillance against tumors are type I invariant natural killer T (NKT) cells, which recognize glycolipid antigens presented by CD1d, an MHC class I-like molecule, expressed also on CLL cells [71,72]. A lower frequency of circulating NKT cells were found in CLL patients compared to healthy controls [72] and were correlated with progressive disease [73]. Indeed, the reduction of NKT-cell number negatively affects antitumor response, as it causes lower TNF expression and a decrease in the production of IFN-γ and cytokines involved in T- and NK-cell activation [74]. In CLL patients, CD1d is expressed on malignant B cells in a lower percentage comparted to normal B cells [73]. However, CD1d expression increases along with disease progression and correlates with unfavorable prognostic factors [i.e., higher ZAP70 and CD38 expression, presence of del(11q) and/or del(17p)] and shorter TTFT and OS [72]. Similarly, Gorini et al. showed in 46 patients with CLL a significant correlation between lower NKT-cell number and higher numbers of malignant CD1d^high^ cells with disease progression [75]. Along with numerical abnormalities, a functional impairment of NKT cells isolated from CLL patients with high CD1d expression was also reported, possibly due to progressive exhaustion of NKT cells following chronic stimulation from CD1d^high^ CLL cells [75]. To date, little is known on how NKT cells can affect therapeutical approaches in CLL, however it has been reported that, even if decreased in number, they are not functionally impaired after chemotherapy [76]. Therefore, therapies aiming at enhancing NKT cells in CLL may be a possible advantageous tool to reinforce antitumor immunity in CLL.

### 2.3. Normal B Cells and Hypogammaglobulinemia

Regarding normal B-cell dysfunctions, most available data focus on immunoglobulin (Ig) deficiency, which results from CLL cells inhibition on the residual subset of normal B cells. Hypogammaglobulinemia is a common condition in patients with CLL, with a frequency ranging from 20% to 70% of cases, depending on the heterogeneity of the analyzed populations [77,78,79,80]. Data regarding the impact of hypogammaglobulinemia on infection rates are controversial. Although several groups established an association between the presence of hypogammaglobulinemia and the occurrence of infections [81,82,83], few reports showed that Ig deficiency does not specifically correlate with the incidence of infections, but rather with an increased risk of deaths from all causes [84,85], thus indicating hypogammaglobulinemia as a marker of leukemia-induced microenvironment alteration and, in general, of disease aggressiveness. In line with this concept, the presence of Ig deficiency was reported to correlate with more advanced stages of disease (i.e., Rai III–IV and Binet B–C) [86,87,88] and with patients’ high-risk features (i.e., unmutated IGHV or unfavorable cytogenetics) [88,89].

The overall impact of hypogammaglobulinemia on the OS of patients with CLL remains unclear. In a cohort of 159 newly diagnosed patients, Andersen and colleagues reported that any type of Ig deficiency is an adverse prognostic factor for OS [85]. Conversely, data from the Israeli CLL study group, including 1113 Binet stage A patients, and from the Mayo Clinic group, including 1482 newly diagnosed patients, described no significant association between hypogammaglobulinemia and OS [88,90]. In terms of Ig classes, IgA deficiency seems to be a strong negative predictor, as shown by the association of reduced IgA levels with shorter TTFT, treatment-free-survival (TFS) and-in some cases-OS [79,89,90,91,92,93]. Interestingly, the ability of ibrutinib administration to improve serum IgA levels – with an observed lower rate of infections in patients showing greater improvements in IgA – further confirmed the role of IgA as both an active weapon against infections and an indicator of improved immune functionality [94]. Concerning the prognostic impact of other Ig classes, decreased IgM levels have been reported to predict shorter TFS and OS [85], while IgG deficiency, and especially low levels of IgG1 and IgG3 subclasses, have been associated with shorter TFS and OS in univariate analysis [87]. The main reason for the controversy of these observations lies in the retrospective nature of most studies, which enrolled heterogeneous and often not comparable patient populations, mainly consisting of early-stage patients. Further prospective studies with a more balanced distribution of patients in terms of disease stage and indications to treatment will be certainly instrumental in the definition of the impact of Ig deficiencies on patients’ outcome.

### 2.4. Myeloid Cells

In conditions of chronic inflammation, monocytes, macrophages and dendritic cells (DCs) differentiate within the inflammatory environment and acquire immunosuppressive characteristics, thus becoming myeloid-derived suppressor cells (MDSCs). This reprogramming process is induced by several factors, some of which derive directly from tumor cells and allow immune escape [95]. In the setting of CLL, the crosstalk between cancer cells and their myeloid microenvironment has been extensively studied. Indeed, patients with CLL present an increased number of CD14+HLA-DR^low^ monocytes [96,97,98]. This correlates—from the biologic standpoint—with decreased antigen-presenting capacity and decreased immune-stimulatory capacity of monocytes [96] and – from the clinical standpoint – with advanced Rai stages (i.e., III and IV) [99]. Moreover, higher monocytic MDSCs (M-MDSCs) count was noticed in patients presenting high-risk disease features [i.e., ZAP70 positivity, presence of del(11q) and/or del(17p), or unmutated IGHV] [99] and correlated with shorter time to progression (TTP), TTFT and OS [97,99,100].

Another key player of myeloid origin in the tumor microenvironment are the nurse like cells (NLCs), which are tumor-associated macrophages exhibiting M2 hallmarks and secreting immunosuppressive and tumor-supportive cytokines [101]. NLCs induce apoptosis-resistance in tumor cells [102], and elevated serum levels of NLCs-secreted cytokines in patients with CLL correlate with poor prognostic factors (i.e., Rai stage III or IV, elevated LDH and/or β2-microglobulin) and have a negative impact on OS [103,104].

Although little is known on how these alterations of the tumor microenvironment can affect treatment results, a recent study has reported on the impact of ibrutinib on the myeloid compartment. Ferrer et al. showed that after treatment initiation, polymorphonuclear MDSCs (PMN-MDSCs) numbers progressively decline, whereas M-MDSC numbers are unaffected. Notably, this ibrutinib-induced reduction in PMN-MDSCs on one hand drove the differentiation of T lymphocytes toward less immunosuppressive Th cells, and on the other hand correlated with a decrease in CLL cell number and with a clinical improvement [100]. Based on these observations, immunotherapeutic strategy lowering the number of MDSCs, and more specifically of PMN-MDSCs, might be a valuable option for treatment of patients with CLL.

### 2.5. Humoral Immunity: Complement and Cytokines

Beside hypogammaglobulinemia, other alterations of the humoral immunity have been broadly described in CLL patients. For instance, exhaustion of the classical pathway of the complement cascade has been reported in 38% of patients, resulting in a hampered complement-dependent cytotoxicity that may affect both protection from infections and the therapeutic efficacy of mAbs [105]. Interestingly, an impact of changes in the complement cascade on CLL prognosis has already been suggested. Indeed, Varga et al. have shown that a low activity of the classical complement pathway at CLL diagnosis predicts a short OS, especially in patients with Rai stage II and III disease [106].

Another peculiar aspect of the altered humoral immunity in CLL is the presence of a specific cytokine signature, capable of influencing the course of the disease [107]. In CLL, cytokines are mainly produced by leukemic cells, but they may also originate from the interactions between the neoplastic clone and immune cells (i.e., T cells, NLCs, bone marrow stromal cells) [108]. The modulation of the cytokine milieu within the tumor can in turn stimulate the growth and survival of the neoplastic clone [109], through the receptor-mediated activation of different signaling pathways involved in cell migration, proliferation and apoptotic function. Over the years, the altered expression of several cytokines has been correlated with CLL biological characteristics and prognostic features (Table 2). Cytokine profiling of patients’ sera has shown that increased levels of Th2-related cytokines and decreased levels of Th1-related cytokines correlate with an aggressive disease and predict the need of therapy [110]. Notably, in line with previous data disclosing a correlation between serum concentration of some chemokines and CLL prognosis [111], Yan and colleagues identified a panel of 17 cytokines/chemokines that were significantly elevated in CLL patients compared to healthy controls [112]. Among these, a group of chemokines specifically involved in cell migration and in T-cell attraction and infiltration have demonstrated to predict a worse prognosis. More recently, in a study by Agarwal and colleagues, six cytokines (i.e., SDF-1/CXCL12, uPAR, IGFBP-2, BMP-4, MCP-4, IL-1 R4/ST2) resulted to be statistically different between initial and advanced stages of the disease [113]. Of those, SDF-1, a chemoattractant for leukemic B cells expressing CXCR4, resulted to be overexpressed in patients with advanced disease [113]. Our group has recently demonstrated that the SDF-1/CXCR4 axis is critically involved in the microenvironment-induced protection of CLL cells through the positive regulation of HIF-1α transcription factor [114]. Interestingly, we also reported that HIF-1α, which regulates tumor cell adaptation to hypoxia and to microenvironmental stimuli, is overexpressed in CLL cells from patients carrying IGHV unmutated genes and *TP53* alterations, who are typically characterized by poor outcome and resistance to therapy [115,116]. Of note, the overexpression of chemokine receptors, such as CXCR4, on CLL cells has prompted the investigation of new potential therapeutic targets. Unfortunately, despite showing significant activity against CLL cells, none of CXCR4-inhibitors has so far been transferred to clinical practice [117,118,119].

## 3. Clinically Meaningful Immune Alterations: The Impact of Autoimmunity, Infections and Second Malignancies on the Prognosis of Patients with CLL

The complex immune alterations characterizing CLL eventually manifest in clinically relevant immune dysfunctions, including autoimmune phenomena and increased risk of infections [138,139,140,141]. Also, this immune dysregulation leads to an increased risk of second malignancies in patients with CLL [142,143]. These additional clinical manifestations are overall the most common complications that affect the course and management of this chronic disease, and they impact on the overall CLL prognosis.

### 3.1. Autoimmune Manifestations

Autoimmune phenomena frequently complicate the clinical course of patients affected by CLL. As reviewed in [141], up to a quarter of patients with CLL may present concomitant autoimmune manifestations, which are primarily autoimmune cytopenias, including autoimmune hemolytic anemia (AIHA), immune thrombocytopenia (ITP), pure red cell aplasia and autoimmune granulocytopenia. Conversely, non-hematological autoimmunity, such as Hashimoto’s thyroiditis, rheumatoid arthritis, vasculitis, bullous pemphigus or acquired angioedema, are undoubtedly rarer.

Multiple immune mechanisms are involved in the pathophysiology of CLL-related autoimmune manifestations, and both humoral and cellular immune dysfunctions may support the development of these complications [141]. Interestingly, autoimmune cytopenias appear to occur most frequently in patients with CLL in advanced clinical stage, whereas non-hematological autoimmune complications are more common in the initial phases of the disease, possibly suggesting an even wider heterogeneity in the pathogenic mechanisms [144,145].

The observed association of autoimmune cytopenias with the disease stage may also be determined by the fact that the Binet and Rai staging systems do not discriminate between bone marrow infiltration and autoimmunity as the cause of anemia or thrombocytopenia [146]. Different groups have shown that the survival for patients with anemia or thrombocytopenia of autoimmune origin is longer than for those with cytopenias attributable to bone marrow infiltration [147,148,149]. Beside the clinical stage, a significant association between CLL-related autoimmune complications and other negative prognostic parameters, such as high lymphocyte count, high β2-microglobulin or LDH level, increased CD38 or ZAP-70 expression, adverse FISH [i.e., del(17p) or del(11q)], or unmutated IGHV has been observed in different cohorts [149,150,151,152,153,154,155,156,157,158]. Of note, not all prognostic parameters were assessed by all groups, and-most importantly-not all publications are concordant in reporting an association between autoimmune manifestations and some of the variables, possibly depending on the heterogeneity of the cohorts evaluated, on the retrospective nature of the studies and on the variability in the criteria used to define autoimmune cytopenias.

In spite of the frequently reported association of autoimmune cytopenias with adverse prognostic factors, the number of articles specifically concluding for a significant impact of autoimmune phenomena on the prognosis of patients with CLL is limited. In their cohort of 473 consecutively diagnosed patients with CLL, Visco and colleagues reported a 7% occurrence of AIHA: there was not a significant difference in terms of OS between patients with or without AIHA, but patients developing AIHA earlier in the disease course (i.e., within 48 months after CLL diagnosis) had a significantly inferior OS compared to those with late-onset AIHA or to those who did not develop AIHA at all [159]. Seven-hundred seventy-seven patients with treatment-naive CLL requiring therapy, who were enrolled in the randomized UK LRF CLL4 trial, were evaluated by Dearden et al. [160]. Among patients who were tested for direct antiglobulin test (DAT) at study entry, 89 (14%) resulted positive, and among those with available information, 77 (10%) developed AIHA during treatment. Both DAT positivity and the development of AIHA were predictive for shorter PFS and OS in this cohort. Accordingly, other groups confirmed the negative prognostic impact of DAT positivity at CLL diagnosis, independently from the occurrence of AIHA [161,162,163].

In a large cohort of 1477 patients with CLL, Shvidel et al. identified 100 patients with autoimmune cytopenia (7%) [164]. As compared with control patients without cytopenia and who never developed AIHA or ITP, patients with AIHA had a significantly worse outcome in terms of OS from the time of CLL diagnosis, whereas the survival was similar for patients with ITP and controls. Conversely, in a cohort of 1278 newly diagnosed patients with CLL, 64 cases of ITP were identified (5%) and the development of the autoimmune phenomenon at any time during the disease course conferred a shorter OS [152]. In these patients, an early occurrence of ITP (i.e., within 24 months after CLL diagnosis) was found to be an independent adverse prognostic factor for OS. As for the association with prognostic parameters, the evaluation of the impact of autoimmunity on OS also produced inconsistent results, mainly due to the heterogeneity of the patient populations analyzed in different studies. Notably, the prognostic impact of non-hematological autoimmune manifestations is even more difficult to ascertain due to the rarity of their occurrence.

It is widely accepted that the initial treatment for CLL-associated autoimmune cytopenias is based on steroids, possibly in combination with rituximab and/or immunosuppressive drugs, whereas when CLL treatment criteria are fulfilled or when the autoimmune phenomenon is not controlled, a CLL-directed treatment is recommended [146,165]. Few data are available regarding the impact of autoimmune manifestations on response to CLL-directed therapy. In the previously mentioned UK LRF CLL4 trial reported by Dearden and colleagues, where patients were randomized to receive chlorambucil, fludarabine or fludarabine plus cyclophosphamide, it was noted that patients who developed AIHA had inferior response rates and quality [160]. Of note, patients with AIHA did receive less therapy. More recently, the presence of a positive DAT was found to be a predictor for non-response to frontline chemoimmunotherapy treatment in a cohort of 120 patients with CLL [166].

The therapeutic scenario for CLL recently changed, with the introduction of targeted agents, such as B-cell receptor inhibitors and Bcl-2 antagonists [167]. Interestingly, data from our group and others indicate that in patients treated with ibrutinib, idelalisib or venetoclax, a pre-existing autoimmune cytopenia does not carry an adverse impact on patients’ prognosis, suggesting that these effective drugs might be able to attenuate the inferior outcome associated with autoimmune manifestations [156,158].

### 3.2. Infections

In patients with CLL, infection is a common cause of morbidity and mortality, accounting for up to more than 50% of the deaths–depending on the characteristics of the patients’ cohorts included in the different studies–and the negative impact of infections raises in patients with multiple comorbidities [85,168,169]. Interestingly, as compared to a control population, the risk of infection is higher not only in patients with CLL, but also in those with MBL, the pre-disease stage preceding CLL [170]. The increased risk of infection is certainly dependent on the underlying CLL-related immune dysfunctions, but also on the immune perturbations specifically related to different CLL-directed therapies. Accordingly, current guidelines recommend antimicrobial prophylaxis in patients with a higher risk of developing infections, based on the treatment regimen received [165].

The possible correlation of infections and CLL prognostic parameters has been explored. Francis at al. retrospectively evaluated a cohort of 280 patients, and–as expected–infection rate inversely associated with Ig levels [81]. Also, patients with advanced clinical stage, unmutated IGHV, genetic abnormalities [*TP53* mutation or deletion, *ATM* mutation or deletion, tris(12)] and those with CD38 positivity had a shorter time-to-first major infection, with both stage and IGHV mutational status maintaining their independent value in multivariate analysis. The same four parameters negatively impacted infection-related mortality, possibly reflecting an association between disease aggressiveness and immune deficiency. Consistently, in the monocentric cohort of 706 patients reported by Visentin and colleagues, major infections–defined as events requiring in patient management or intravenous antibiotics–were associated with clinical stage, IGHV mutational status, high-risk cytogenetics and CD38 positivity [80]. More recently, Andersen and colleagues interrogated the Danish National CLL registry, assessing a cohort of 2905 patients diagnosed with CLL between the years 2008 and 2016 [82]. In multivariate analysis, variables that significantly associated with infection-specific hazard rates were Binet stage, β2-microglobulin and IgA levels. As highlighted by the authors, the lack of association of the risk of infections with adverse FISH abnormalities or unmutated IGHV status might be explained by the impact of these variables on the probability of treatment, which was a competing risk in this model.

Undoubtedly, the development of models capable to identify patients more susceptible of developing severe infections during the course of their disease would be extremely useful, also in terms of treatment selection. To this aim, Agius and colleagues recently developed a machine learning model identifying with high precision patients at risk of severe infection within 2 years of CLL diagnosis [171].

Regarding the evaluation of the impact of infections on the overall prognosis of patients with CLL, in the already cited Danish registry cohort, patients with CLL who have had an infection during the first year after diagnosis–identified as those who had a blood culture drawn prior to CLL therapy–had a significantly shorter TFS and OS [172]. In their monocentric cohort of 706 patients, Visentin and colleagues showed that patients with a history of major infection had a shorter OS compared to those who did not experience this complication, and this factor maintained its impact on survival in multivariate analysis [80]. Data from Crassini et al., who analyzed the long-term follow-up (9.5 years) of a cohort of 147 patients, confirmed a significant association between the occurrence of serious or recurrent infectious complications within the first year of observation and a shorter OS [173].

It needs to be highlighted that in the last few years, the introduction of targeted agents for the treatment of patients with CLL may have had an impact on the infectious complication spectrum for these patients (reviewed in [140]). For example, opportunistic pathogens have been recognized as an emerging cause of infection in patients treated with ibrutinib. However, the infectious risk seems more related to the disease itself and to the previous treatment status than to the administered drugs. Recently, Mauro and colleagues specifically evaluated the prognostic impact of infections in 494 patients with CLL treated with ibrutinib [174]. Ibrutinib was permanently discontinued in 9% of patients due to infections and OS for patients who had a severe infection or pneumonia was significantly shorter compared to those infection-free.

Finally, it is worth to be mentioned that in the current worldwide pandemic scenario, patients with CLL can be severely affected by SARS-CoV-2 infection [175]. Older patients seem to be at increased risk of infection, with a high incidence of mortality among hospitalized patients (reviewed in [176]). Interestingly, it has been postulated that BTK inhibitors such as ibrutinib or acalabrutinib could attenuate hyperinflammatory responses thus exerting protection against severe disease course, but to date data are still controversial and prospective clinical trials are ongoing [176]).

### 3.3. Second Malignancies

Considering the high median age at diagnosis (i.e., 70 years) of patients with CLL [177], who mostly remain under surveillance for years, it is not surprising how during the course of the disease the onset of another cancer often occurs, and has a considerable impact on the patient’s prognosis. Strati et al. reported their results on a prospective cohort of 1143 newly diagnosed patients with CLL, amongst whom 225 deaths were reported after a median follow-up of 6 years: cause of death was attributed to a second malignancy in 19% of patients [168]. Undoubtedly, together with the age, CLL-related factors–such as the loss of immune surveillance and previous treatments–exert their contribute on the occurrence of other malignancies. Registry-derived data from the early 1990s already reported a significantly increased risk of developing a second malignancy in patients with CLL, compared to the general population [178].

The contribution of previous treatments in this increased risk has been specifically evaluated by Falchi and colleagues, who analyzed the impact of other malignancies in 797 patients with CLL who survived >10 years [143]. In this cohort, an excess of cancer diagnosis in patients with CLL compared to the general population was confirmed, with a standardized incidence ratio (SIR) of 1.2. Interestingly, the cumulative frequency of other cancers was similar in patients who received treatment for CLL and in those who remained untreated (36%), and the therapy for CLL was not associated with the occurrence of other malignancies in multivariate analysis. Conversely, a large population-based analysis on data from 38754 patients with CLL, derived from the Surveillance Epidemiology and End Results (SEER) database, showed an increased risk of second primary malignancies in patients who had received prior chemotherapy compared to those untreated or with an unknown treatment status (SIR 1.38 vs. 1.16) [179]. However, when the analysis was restricted to patients diagnosed with CLL in the time interval 2003–2015–when an overall increasing trend of second primary malignancies was noted–no difference was found in the risk for solid tumors between previously treated and untreated patients.

Besides the impact of CLL-directed treatments, some attempts have been made to identify a possible correlation between the development of second cancers and CLL-related biological parameters. In their retrospective analysis on 2028 patients with CLL, Tsimberidou and colleagues reported that elevated levels of β2-microglobulin and LDH, but not the presence of cytogenetic aberrations, were independent factors predicting for the development of second cancers [180]. However, in the already cited analysis on long-term survivors performed by Falchi et al., β2-microglobulin did not emerge as independent predictor, possibly reflecting differences in the patients’ cohorts [143]. Additional studies should assess the impact of risk factors, including immune parameters, on the risk of developing second primary malignancies.

The group at Mayo Clinic specifically focused on the risk of developing a second lymphoproliferative disorder, retrospectively analyzing 962 patients with CLL [181]. After a median follow-up of 3.3 years, 2.9% of patients developed a second lymphoproliferative disorder, and this was not associated with CLL biological characteristics, such as ZAP70 or CD38 expression, IGHV mutational status or cytogenetic aberrations. The incidence of and risk factors for second malignancies was also recently evaluated in a cohort of 691 patients treated with ibrutinib or acalabrutinib [182]. After a median follow-up of 44 months, 20% of patients were diagnosed with a non-melanoma skin cancer and 9% with other primary malignancies, which were responsible for 13% of deaths. In this cohort, the SIR for secondary invasive cancers was 2.2, and a lower risk for other cancers was significantly associated in multivariate analysis with a higher baseline CD8+ T-cell count, supporting a correlation between immune function and carcinogenesis in this patient population.

It is conceivable that the presence of another malignancy may impact the overall prognosis of a patient. In the previously cited analysis from Tsimberidou et al., patients who had a history of a prior malignant neoplasms at the time of presentation with CLL had a significantly shorter OS as compared to those who did not. However, the authors did not specifically evaluate the survival of patients who developed a second malignancy after CLL diagnosis compared to those who did not [180]. Among 12,041 patients with CLL from the Swedish Cancer Registry, Toro and colleagues reported 236 cases of non-melanoma skin cancer, including 111 squamous cell cancers [183]. These patients had a significantly shorter OS than CLL patients without non-melanoma skin cancer, and 44% of deaths were attributed to CLL. However, it has to be highlighted that the median age at CLL diagnosis for patients with a non-melanoma skin cancer was significantly higher compared to those without a history of non-melanoma skin cancer (78.5 vs. 71 years). Finally, Royle et al. reported in their cohort of 13580 patients diagnosed with CLL in Australia between 1983 and 2005 a SIR for second primary cancers of 2.17 [184]. Sixty-five% of deaths were attributed to cancer (15% excluding lymphoproliferative neoplasms). Overall, CLL patients had a 2.5 times higher mortality rate as compared to the general population, and their age standardized cancer mortality ratios–excluding lymphoproliferative neoplasms–was 1.72. Additional studies are currently needed to elucidate whether second primary malignancies have a more aggressive evolution, ultimately leading to a worse outcome, in patients with CLL compared to the general population.

## 4. Conclusions and Perspectives

The evaluation of the multiple immune alterations occurring in patients with CLL, which affect both the innate and adaptive immunity, is certainly fundamental to better understand the biology of the disease. Some of these immune system dysfunctions are associated with other disease-specific prognostic hallmarks and have been shown to have a prognostic impact. These observations support the notion that the clinical heterogeneity that characterizes CLL depends not only on the intrinsic features of the tumor clone, but also on the complex interrelation occurring between the cancer cells, the immune system and the tumor microenvironment.

The disease-related immune dysfunctions can be further exacerbated by the immunosuppressive effects of the CLL-directed therapies, constituting a double-hit process that favors the development of clinically relevant manifestations such as autoimmunity, infections and second malignancies. These manifestations may have an impact on the overall patients’ prognosis, not only because they directly impact the clinical outcome, but also because they easily interfere with the treatment program, causing delays or interruptions. Of note, in some patients, autoimmune phenomena, infections or second cancers can even occur simultaneously, on one hand because of a higher susceptibility linked to the intrinsic features of higher-risk subtypes of the disease, and on the other hand because of the mutual influence that these three complications can exert on each other: in patients with autoimmune cytopenia, infections are a frequent complication following immunosuppressive treatments, and second malignancies have been reported as the leading cause of death [147]. The use of less toxic treatments, exerting a reduced impact on the immune system functionality, may be beneficial on the overall management of these patients, especially when other comorbidities are present.

Most of the data presented in this article were collected from the chemotherapy or chemoimmunotherapy era, and the advent of targeted agents may arguably result in changes in the current scenario. Therapies with an improved efficacy prolong patients’ survival and confer a longer follow-up duration, thus increasing the possibility to detect disease-related complications over a longer period of time. Targeted drugs—which along with their anti-tumor activity exert multiple off-target effects on different components of the immune system and tumor microenvironment (reviewed in [1,2,185,186])—may also directly impact on the risk of immune dysfunctions and immune-dependent clinical manifestations. Therefore, we expect that the current wide use of these compounds in the clinical practice and the progressively growing follow-up duration will provide more data and pieces of information to be added to this complex scenario.

## Figures and Tables

**Table 1:** Relevant qualitative NK-cell aberrations reported in patients with CLL.

NK-Cell Aberrations	Consequence	Impact on Disease	Ref.
Reduction of NKp30 and NKp46 activating receptors	Exhaustion state on NK cells	Immune escape	[63]
Increased expression of Tim-3 immune checkpoint	Exhaustion state on NK cells	Immune escape	[63]
Abundance of immature CD56^bright^ NK cells	Reduced NKG2D activating receptor and cytokine (IL-10 and IL-13) secretion	CLL cells survival and proliferation	[69,70]
Irregular NKG2AR activity and reduced killer Ig-like receptors (KIRs)	Hampering of NK-cell cytotoxicity and viability	Compromised immune system	[1]
Lower natural cytokine receptors (NCRs) expression		Immune escape	[69]
NKG2D downregulation	Hampering of NK cytotoxicity		[65]

**Table 2:** Alterations of cytokine levels and their correlation with biological characteristics and/or prognosis in patients with CLL.

Cytokines	Alteration Compared to Healthy Controls	Correlation with Biological Characteristics and/or Prognosis	Ref.
sCD8	Increased	Active disease, advanced Rai stage.	[120]
sCD27	Increased	Advanced Rai stage, elevated β_2_-microglobulin.	[121]
sIL-2R	Increased	Active disease, advanced Rai stage, high lymphocyte count.	[120]
IL-6	Increased	Advanced Rai stage, previous treatment, elevated β_2_-microglobulin, elevated LDH, worse OS *.	[104]
	Increased	Advanced Binet stage, previous treatment, non-CR status, presence of del(17p)/del(11q), shorter absolute LDT, worse TTFT, worse PFS.	[122]
	Increased	Elevated β_2_-microglobulin. IL-6, IL-8 and TNFα levels correlated with each other. In patients ≥ 70 years, IL-6 is a better prognostic marker than IGHV mutational status.	[123]
IL-8	Increased	Active disease (progression from Binet stage A to B/C).	[124]
	Increased	Elevated β_2_-microglobulin. IL-8, IL-6 and TNFα levels correlated with each other.	[123]
	Increased	Advanced Rai stage, elevated β_2_-microglobulin.	[121,125,126]
IL-9	Increased	Advanced stage, elevated β_2_-microglobulin, higher ZAP70 expression.	[127]
	Increased	Advanced Rai stage, higher ZAP70 and CD38 expression.	[128]
IL-10	Increased	Advanced Rai stage, previous treatment, elevated β_2_-microglobulin, elevated LDH, worse OS *.	[104]
	Decreased	Active disease.	[129]
	Increased	High-risk and active disease. Worse TFS (in high-risk group, regardless of IGHV mutational status), worse OS ^§^.	[130]
	Increased	Advanced Rai stage, elevated β_2_-microglobulin.	[121]
IL-23R	Decreased	Worse prognosis in early stage CLL, worse TTFT.	[131]
TNFα	Increased	Advanced stage, elevated β_2-_microglobulin, higher CD38 expression, presence of del(11q), tris(12), chromosome 17 aberrations, worse OS.	[132]
	Increased	High-risk and active disease. Worse TFS (in high-risk group, regardless of IGHV mutational status), worse OS ^§^.	[130]
	Increased	Elevated β_2_-microglobulin. IL-6, IL-8 and TNFα levels correlated with each other. In patients ≥ 70 years, IL-6 is a better prognostic marker than IGHV mutational status.	[123]
SDF-1 and uPAR	Increased	Advanced stage.	[113]
SDF-1 and CXCR4	Increased	Advanced Rai stage.	[133]
IGFBP-2, BMP-4, MCP-4	Decreased	Advanced stage.	[113]
CCR7	Increased	Advanced Rai stage.	[133]
CXCR3	Decreased	Advanced stage, higher CD38 expression, unmutated IGHV status, worse OS.	[134]
CX3CL1	Increased	Lymph node involvement, worse TTT, high risk of progression (especially in earlier stages of disease).	[135]
	Increased	Higher ZAP70 expression.	[136]
CCL3/MIP-1α	Increased	Advanced stage, higher CD38 and ZAP70 expression, unmutated IGHV status.	[137]

* IL-6 and IL-10 emerged as independent prognostic factors for OS both when analyzed individually and in combination. ^§^ The cytokine low-risk group comprised patients with either low TNFα or low IL-10 or those with only one elevated parameter. The cytokine high-risk group comprised patients with both high TNFα and high IL-10.

## Abbreviations

CLL	chronic lymphocytic leukemia
MBC	monoclonal B cells
TEMRA	terminally differentiated memory T cells
EM	effector memory T cells
Th	T helper
Tregs	T regulatory cells
NK	natural killer
NKT	natural killer T
M-MDSCs	monocytic myeloid-derived suppressor cells
NLCs	nurse like cells
DCs	dendritic cells
TTFT	time-to-first treatment
OS	overall survival
PFS	progression-free survival
FCR	fludarabine-cyclophosphamide-rituximab
IGHV	immunoglobulin heavy chain variable region
TCR	T cell receptor
MBL	monoclonal B cell lymphocytosis
mAb	monoclonal antibody
ADCC	antibody-dependent cell-mediated-cytotoxicity
KIRs	killer Ig-like receptors
NCRs	natural cytokine receptors
Ig	immunoglobulin
TFS	treatment-free survival
TTP	time to progression
PMN-MDSCs	polymorphonuclear myeloid-derived suppressor cells
AIHA	autoimmune hemolytic anemia
ITP	immune thrombocytopenia
DAT	direct antiglobulin test
SIR	standardized incidence ratio
SEER	surveillance epidemiology and end results
CR	complete remission
LDT	lymphocyte doubling time
TTT	time-to-treatment
